# Cyclooxygenase 1 mRNA expression is undetectable in Madin Darby Canine Kidney cells

**DOI:** 10.1186/s13104-015-1049-4

**Published:** 2015-03-24

**Authors:** Guillaume Pelletier, Bhaja K Padhi

**Affiliations:** Hazard Identification Division, Environmental Health Science and Research Bureau, Health Canada, Environmental Health Centre, 50 Colombine Driveway, P.L. 0803B, Tunney’s Pasture, Ottawa, Ontario K1A 0L2 Canada

**Keywords:** Madin Darby Canine Kidney cells, Cyclooxygenase, Gene expression

## Abstract

**Background:**

Madin Darby Canine Kidney (MDCK) cells form polarized epithelium *in vitro* and are routinely used in research fields ranging from protein trafficking to influenza. However, the canine origin of these cells also means that compared to man or mouse, genomic resources are more limited and performance of commercially available antibodies often untested. The synthesis of pro-inflammatory prostaglandins in the kidney is mediated by the constitutively expressed cyclooxygenase 1 and the inducible cyclooxygenase 2 (COX-1 and COX-2, respectively). There are conflicting reports on the expression of COX-1 and COX-2 in MDCK cells and this lingering uncertainty about such important pharmacological targets may affect the interpretation of results obtained from this cell line.

**Results:**

In order to definitively settle the issue of cyclooxygenase expression in MDCK cells, we designed PCR primers based on dog genomic sequences to probe *COX-1* and *COX-2* mRNA expression in MDCK cells and dog kidney. We report that while *COX-1* and *COX-2* genes are both expressed in dog kidney, *COX-1* expression is undetectable in MDCK cells.

**Conclusions:**

By improving the characterization of cyclooxygenase expression in MDCK cells, this study will contribute to a better understanding of the properties of this cell line and lead to improved experimental designs and data interpretations.

## Background

Cyclooxygenases are important pharmacological targets, as they are involved in the synthesis of pro-inflammatory prostaglandins [1]. In addition to the constitutively expressed cyclooxygenase 1 (COX-1), the inducible cyclooxygenase 2 (COX-2) is also robustly expressed in the kidney where it plays a role in cellular adaptation to osmotic stress [2,3].

Madin Darby Canine Kidney (MDCK) cells are easy to grow and can form polarized epithelium *in vitro*. These cells have been used notably in investigations on membrane permeability and tight junctions, protein trafficking, influenza viral infection [4] and phorbol ester-induced inflammatory processes [5]. In the decade or so between identification of the inducible *COX-2* gene [6] and sequencing of dog cyclooxygenases [7-10], studies on their expression in MDCK cells assessed COX-1 and COX-2 protein levels by Western blot [2,11-14] and mRNA levels by Northern blot or RT-PCR [11,13,15,16], see Table 1. These studies which were based on antibodies raised against cyclooxygenases from other organisms or on orthologous nucleotide sequences from different species reached conflicting conclusions.

**Table 1:** **Summary of cited studies on the expression of COX-1 and COX-2 in MDCK cells**

**Reference**	**Summary of findings**
Yang et al. [2]	Western blot: COX-2 observed in control MDCK cells and strongly induced by hyperosmolarity. COX-1 expression was not detected in MDCK cells.
Schaeffers et al. [11]	Western blot: COX-1 and COX-2 not detected in MDCK cells.
	Northern blot (mouse probe): *COX-1* and *COX-2* not detected in MDCK cells.
	RT-PCR (based on consensus sequence in mouse, rat and man): Lowly expressed *COX-2* in control MDCK cells is induced by TPA exposure. *COX-1* expression not detected in control and TPA-treated MDCK cells.
Sciorra and Daniel [12]	Western blot: Low COX-1 and COX-2 levels in Control MDCK cells and strong COX-2 (but not COX-1) induction following TPA exposure.
Ostrom et al. [13]	Western blot: COX-1 and COX-2 expressed in MDCK cells, but not induced by a variety of factors, including TPA.
	RT-PCR (based on conserved sequence across species): *COX-2* transcript amplified, but not *COX-1* (despite numerous attempts).
Benitah et al. [14]	Western blot: COX-1 and COX-2 both detected in control MDCK cells.
Cowley et al. [15]	Northern blot (mouse probe): *COX-2* transcript present in control MDCK cells and quickly induced by hyperosmolarity. *COX-1* not detected in control MDCK cells but weak signal detected following chronic hyperosmolarity. (Cross-hybridisation of cyclooxygenase probes was observed.)
Kay-Mugford et al. [16]	Northern blot (human probe): *COX-1* and *COX-2* detected in MDCK cells.
	RT-PCR (primers based on human sequence): *COX-2* expression not detected in MDCK cells.
Knottenbelt et al. [17]	Western blot: COX-1 and COX-2 not detected in MDCK cells.
Reyes-Martin et al. [18]	Western blot: COX-1 and COX-2 both detected in control MDCK cells and strongly induced following H_2_O_2_ exposure.
Flores-Benitez et al. [19]	Western blot: COX-1 and COX-2 both detected in control MDCK cells, but not induced following EGF treatment.
Steinert et al. [20]	Western blot: COX-2 expressed in control MDCK cells and induced by hyperosmolarity.
	Northern blot (human probe): *COX-2* expressed in control MDCK cells and induced by hyperosmolarity.

In order to definitively settle the issue of *COX-1* and *COX-2* expression in MDCK cells, we designed PCR primers based on published dog genomic and mRNA sequences retrieved from ENSEMBL (www.ensembl.org) and NCBI (www.ncbi.nlm.nih.gov) databases for the Prostaglandin G/H synthase 1 and 2 genes (*PTGS1* and *PTGS2*) coding for the COX-1 and COX-2 proteins, respectively. These primers were then used for PCR amplification of mRNA and genomic DNA from MDCK cells and dog kidney.

## Results

**Figure 1:**
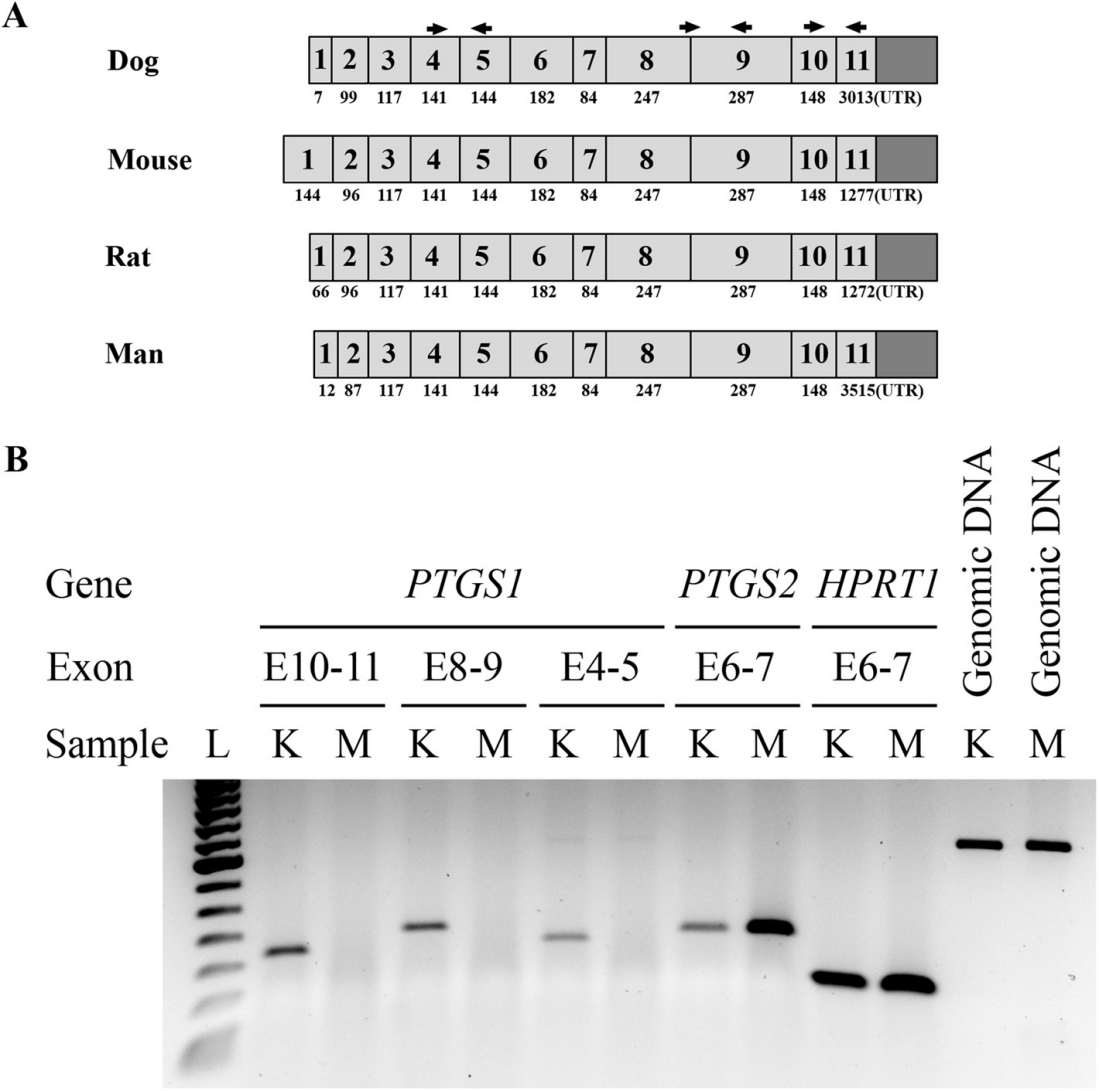
**Expression of cyclooxygenases in MDCK cells and dog kidney. A)** Schematic representation of the exonic structure of the *PTGS1* transcript (coding for the COX-1 protein) in dog, mouse, rat and human. Exon structure and alignment were based on NCBI RefSeq NM_001003023, NM_08969, NM_017043 and NM_001271162, accessed on February 6, 2015. Exons are numbered and their lengths indicated along with the locations of the PCR primers used in this study. Exons are not all shown to scale. **B)** Representative gel showing the expression of *COX-1* (*PTGS1*), *COX-2* (*PTGS2*) and *HPRT1* in MDCK cells (M) and dog kidney (K). Three regions of the *PTGS1* gene, namely Exon 10-11 (E10-11), E8-9 and E4-5 were targeted for PCR amplification. The Cox1_E4-5 primers were also used to amplify genomic DNA from MDCK cells and dog kidney, generating a larger amplicon (380 bp) due to the inclusion of a 212 bp intron. A 50-bp DNA ladder (L) is shown on the left.

The expression of *COX-1* (*PTGS1*)*, COX-2* (*PTGS2*) and *HPRT1* (hypoxanthine phosphoribosyltransferase 1) genes in MDCK cells and dog kidney were assessed under similar experimental conditions. As expected, primers for the housekeeping gene *HPRT1* and *COX-2* amplified their target sequences from both dog kidney and MDCK cells (Figure 1B). However, while the three primer sets for the *COX-1* gene successfully amplified their target sequences from dog kidney mRNA, they failed to amplify mRNA from MDCK cells (Figure 1B). Further PCR investigation using *Cox1_E4-5* primers (Table 2) to probe genomic DNAs amplified a 380 bp fragment (including 212 bp of *COX-1* intronic sequences) from both dog kidney and MDCK cells (Figure 1B). Hence, although *COX-1* mRNA is not detected in MDCK cells, *COX-1* coding sequences are present in MDCK genome and the exact reason behind this lack of expression remains to be determined.

**Table 2:** **Primer sequence, location and expected amplicon sizes for dog** ***COX-1*** **(** ***PTGS1*** **),** ***COX-2*** **(** ***PTGS2*** **) and** ***HPRT1*** **genes**

**Gene**	**Accession**	**Primer name**	**Primer sequence**	**Amplicon (bp)**	**Location**
*PTGS1*	NM_001003023	Cox1_E10-11 F	TGTCCTTCCAGGAACTCACA	161	Exon10
		Cox1_E10-11R	GAAGGGAGCCCCAATTTCTA		Exon11
*PTGS1*	NM_001003023	Cox1_E8-9 F	ATCCTCATTGGGGAGACCAT	193	Exon8-9
		Cox1_E8-9R	AACCCACCCAGAAGGAGTCT		Exon9
*PTGS1*	NM_001003023	Cox1_E4-5 F	CTTTCCTCCACTTCCTGCTG	168	Exon4
		Cox1_E4-5R	AGAAGGACTCCCAGCTGATG		Exon5
*PTGS2*	NM_001003354	Cox2_E6-7 F	GTTCATTCCTGATCCCCAAG	186	Exon6
		Cox2_E6-7R	TTGAAAAGGCGCAGTTTATG		Exon7
*HPRT1*	NM_001003357	Hprt1_F	TGACACTGGGAAAACAATGCAGACT	110	Exon6
		Hprt1_R	AGCCAACACTTCGAGGGGTCCT		Exon7

## Discussion

Several research groups assessed COX-1 and COX-2 protein levels in MDCK cells by Western blot using antibodies raised against protein epitopes from different mammalian species, often leading to conflicting results [2,11-14,17-20], see Table 1. Although the commercial suppliers of the antibodies were generally mentioned, this information was not always sufficient to identify the specific antibody used. This ambiguity on antibody identities, along with the sometimes incomplete description of the MDCK strain used [4,21], hampered comparison between these studies.

Prior to the availability of dog cyclooxygenase gene sequences, detection of *COX-1* and *COX-2* mRNA expression in MDCK cells relied on Northern blot using orthologous human or mouse sequences as probes. These investigations also reached differing conclusions [11,15,16] which may be explained by non-specific cross-species hybridisation of the probes. Efforts to assess *COX-1* gene expression in MDCK cells by RT-PCR proved unsuccessful [11,13]. Although aberrant cyclooxygenase expression in MDCK cells was suggested [16], incomplete knowledge of the dog genome at that time prevented a definitive conclusion.

In this report, our attempts to amplify *COX-1* mRNA from MDCK cells using three different sets of PCR primers designed from dog genomic sequences and covering different regions of the *COX-1* gene (Figure 1A) also failed (Figure 1B). The same primer sets were successfully used to amplify their respective targets from dog kidney mRNA, as confirmed by sequencing of the amplicons generated. In light of a report on COX-1 protein induction in MDCK cells [18], we also assessed *COX-1* expression in MDCK cells exposed to 12-O-tetradecanoylphorbol-13-acetate (TPA) as described in [22], failing again to detect any expression (data not shown). It is theoretically possible that the relatively long half-life of COX-1 protein [23] results in the presence of a residual level of this enzyme despite undetectable gene expression. However, our failure to detect *COX-1* mRNA after 35 PCR amplification cycles suggests at best an infinitesimal expression and questions the biological plausibility of a significant role for this enzyme in the synthesis of pro-inflammatory prostaglandin mediators by MDCK cells.

Despite the use of multiple primer sets (to prevent false negative results due to faulty primers or alternative splicing), the inclusion of a positive control for *COX-1* gene expression (dog kidney mRNA), PCR amplifications conducted for up to 35 cycles (to ensure detection of minute expression), confirmation of amplification specificity (by sequencing of the amplicons), and additional verifications of its inducibility (following induction of prostaglandin synthesis in TPA-treated cells), we were consistently unable to detect *COX-1* gene expression in MDCK cells. Hence, we reach the same conclusion as older RT-PCR investigations predating sequencing of dog *COX-1* and *COX-2* genes [11,13]. Our survey of curated dog microarray datasets at NCBI’s Gene Expression Omnibus database (www.ncbi.nlm.nih.gov/geo, accessed on February 13, 2014) also revealed that while *COX-1* and *COX-2* expression is detected in several dog tissues (for example, see dataset GDS4164), *COX-1* is flagged as absent in MDCK cells (dataset GDS3267).

## Conclusions

We conclude that in absence of detectable *COX-1* gene expression, prostaglandin synthesis and associated inflammatory processes in MDCK cells are mediated by the inducible *COX-2* gene. However, *COX-1* gene expression in MDCK cells is sometimes assumed and the specific COX-1 inhibitor SC-560 used [3,13,18-20,24]. Our results clearly suggest that effects of this inhibitor in MDCK cells are most likely independent of COX-1 inhibition [25]. This improved characterization of cyclooxygenase expression in MDCK cells provides new insights into the biology of this cell line and will contribute to better experimental design and enhanced interpretation of the results obtained.

## Methods

### Reagents and cell culture

The MDCK (NBL-2) cell line was purchased from American Type Culture Collection (ATCC, Manassas, VA, USA) in 2002, grown for a few passages and then cryopreserved in liquid nitrogen. MDCK cell aliquots used in this study were sub-cultured for a maximum of 15 passages. Cell culture media and fetal bovine serum were also purchased from ATCC. MDCK cells were grown in Eagle Minimum Essential Medium with Earle’s Balanced Salt Solution, 10% fetal bovine serum and 1% penicillin-streptomycin (Life Technologies, Burlington, ON, Canada) in a humidified incubator at 37°C and 5% CO_2_. MDCK cells seeded at 70,000 cells/cm^2^ were allowed to grow for two days before nucleic acid isolation.

### Total RNA and genomic DNA isolation

Total RNA from MDCK cells was isolated and purified using the RNeasy kit (Qiagen, Toronto, ON, Canada). Dog kidney total RNA was purchased from Zyagen (San Diego, CA, USA), and further purified by RNeasy kit after DNase treatment to avoid genomic DNA contamination. RNA was quantified using a Nanodrop 1000 spectrometer (Thermo Scientific, Waltham, MA, USA) and its integrity assessed using a 2100 Bioanalyzer (Agilent Technologies, Mississauga, ON, Canada). Dog genomic DNA was obtained from Zyagen. MDCK genomic DNA was purified using Flexi Gene DNA kit (Qiagen).

### Primer design and PCR amplification

Approaches for primer design and PCR amplification were previously described [26]. Briefly, primers were designed from dog *PTGS1* (*COX-1*), *PTGS2* (*COX-2*) and hypoxanthine phosphoribosyltransferase 1 (*HPRT1*) nucleotide sequences retrieved from the NCBI Refseq database (Table 2). Two micrograms of total RNA were used for first strand cDNA synthesis, using Superscript III™ reverse transcriptase (Life Technologies) and one-fifth of the cDNA reaction or 100 ng genomic DNA was used for gene expression analyses. PCR reactions were carried out for 35 amplification cycles (30 s denaturation at 94°C*,* 30 s annealing at 60°C and 60 s extension at 72°C). *PTGS1* and *PTGS2* amplification products were assessed on agarose gels and sequenced to confirm their identities. Gene expression assessment was performed on at least 4 different control RNA samples from another investigation using MDCK cells [22], while assessment of MDCK genomic sequence was performed on 5 different genomic DNA samples.

## Abbreviations

*COX-1*: Cyclooxygenase 1
*COX-2*: Cyclooxygenase 2
EGF: Epidermal Growth Factor
*HPRT1*: Hypoxanthine phosphoribosyltransferase 1
MDCK: Madin Darby Canine Kidney
*PTGS1*: Prostaglandin G/H synthase 1
*PTGS2*: Prostaglandin G/H synthase 2
RT-PCR: Reverse transcription-polymerase chain reaction
TPA: 12-O-tetradecanoylphorbol-13-acetate
